# Rupture of Bilateral Theca Lutein Cysts During Pregnancy: A Case Report

**DOI:** 10.7759/cureus.29758

**Published:** 2022-09-29

**Authors:** Sowjanya Kurakula, Vandana Muralidharan, Lohith Chengappa Appaneravanda, Navya N, Gayathri K B

**Affiliations:** 1 Obstetrics and Gynecology, Gandhi Medical College, Musheerabad, IND; 2 Obstetrics and Gynecology, Sekgoma Memorial Hospital, Serowe, BWA; 3 Obstetrics and Gynecology, Nyangabgwe Referral Hospital, Francistown, BWA; 4 Obstetrics and Gynecology, Mamta Institute of Medical Sciences, Khammam, IND; 5 Obstetrics and Gynecology, Lifeline Medical Associates, New Jersey, USA; 6 Embryology, Gunasheela Surgical and Maternity Hospital, Bangalore, IND; 7 Fetal Medicine, Ovum Hospital, Bangalore, IND; 8 Obstetrics and Gynecology, Dr. Pinnamaneni Siddhartha Institute of Medical Sciences & Research Foundation, Vijayawada, IND

**Keywords:** adnexal mass in the first trimester, cyst rupture, theca lutein cysts during pregnancy, hyperreactio luteinalis, case report

## Abstract

Hyperreactio luteinalis is a rare condition characterized by the presence of bilateral theca lutein cysts, which occur rarely in a singleton normal pregnancy. Bilateral multicystic ovarian enlargement occurs due to elevated beta-human chorionic gonadotropin. We present a rare case of a 23-year-old primigravida in her tenth week of gestation, presenting with an acute abdomen, with increasing abdominal girth, diagnosed with a rupture of theca lutein cyst, and posted for laparotomy. Theca lutein cyst doesn’t affect the course of pregnancy and has a spontaneous regression after delivery. Our case is different due to the occurrence of cysts in a singleton pregnancy and the rupture of these cysts during pregnancy. Unless complicated by torsion, rupture, or hemorrhage, most theca lutein cysts are managed conservatively.

## Introduction

Theca lutein cysts in pregnancy are asymptomatic, self-limiting, and are diagnosed incidentally during ultrasonography or cesarean section. It is uncommon for them to occur in an uncomplicated pregnancy. The prevalence of adnexal masses during pregnancy has been around 1% to 4% [1]. Functional ovarian cysts during pregnancy are of three varieties, which include follicular cysts, corpus luteal cysts, and theca lutein cysts. Of them, theca lutein cysts are the least common. The conditions associated with hyperreactio luteinalis include hydatidiform mole, choriocarcinoma, multiple pregnancies, erythroblastosis fetalis, diabetes complicating pregnancy, clomiphene intake, ovulation induction with gonadotropin analogs, non-immune hydrops fetalis, and normal pregnancy [2]. In hyperreactio luteinalis, ovaries can massively enlarge to a volume of 1500 ml, with an average diameter of the ovary found to be 15 cm [3]. Often, expectant management is suggested in asymptomatic cases.

## Case presentation

A 23-year-old primigravida, who had a spontaneous conception and was at 10 weeks of gestation, was admitted to the inpatient department with diffused abdominal pain for the past week, increasing in intensity by the day. The pain was stretching in nature, starting in the lower abdomen and progressing to the whole of the abdomen. The patient rated the pain as 8/10 on the pain scale with no aggravating or relieving factors. The pain was associated with nausea and two episodes of vomiting. The patient had never received any sort of hormonal treatment in the past. There was no significant past medical, surgical, or treatment history.

When her vital statistics were recorded, her temperature was normal, her pulse rate was 110 beats per minute, her blood pressure was 110/70 mm of mercury, and her weight was 45 kg. Upon examination of the heart, S1 and S2 sounds were heard, but there were no murmurs. Bilateral vesicular breath sounds were heard in the lungs. On physical examination, it was noticed that the patient was thin-built and poorly nourished. Her review of organ systems revealed no abnormalities. The patient was found to have an abdominal mass of 24 weeks' size with ill-defined borders, tenderness, and guarding present. Shifting dullness was present. Her pelvic examination disclosed bilateral fornices fullness with mild tenderness; the fundus was not palpable and the cervix was soft and uneffaced.

The blood report showed that hemoglobin was 10.8 gm/dL, hematocrit was 30%, mean corpuscular volume was 80 µm, platelet counts were normal, urinalysis was within normal limits, random blood sugar was 88 mg/dL, blood group was O+ve, rapid plasma reagin was negative, and she was seronegative for human immunodeficiency virus and hepatitis surface antigen. The ultrasonography was suggestive of a single viable fetus of 10 weeks of gestation by a crown-rump length with huge ovarian multi-loculated bilateral cysts 175 x 120 mm on the right and 168 x 130 mm on the left ovaries. There was a moderate amount of free fluid in the lower abdomen and pelvis and even in Morison's pouch.

**Figure 1:**
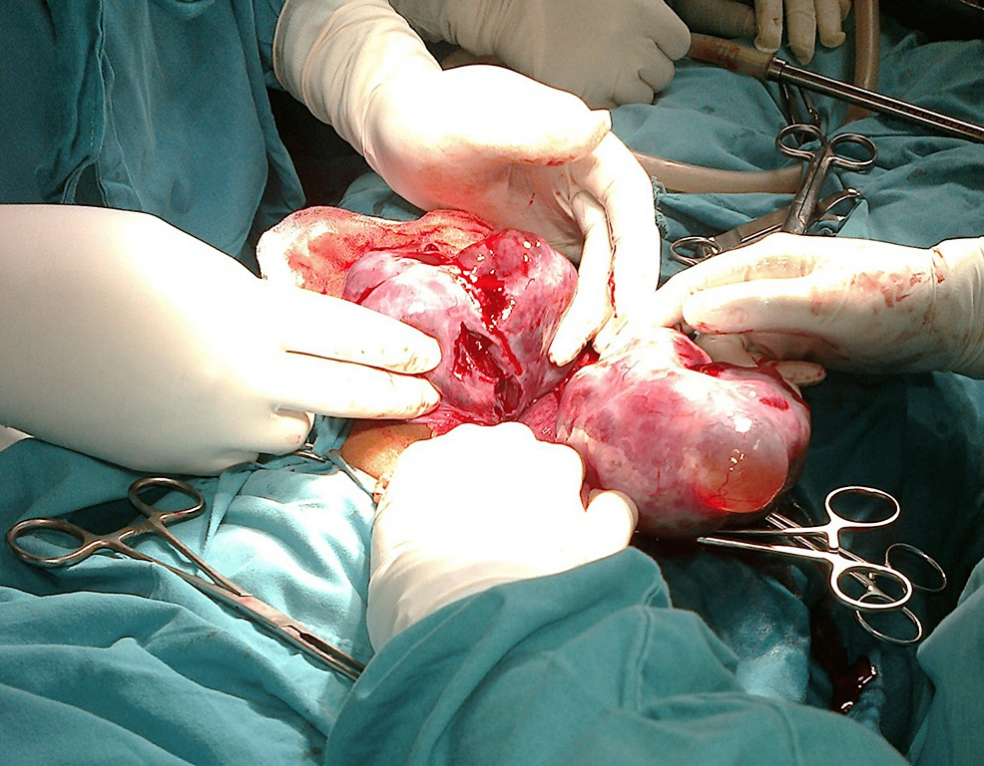
Bilateral theca lutein cysts with spontaneous rupture in multiple areas were noted.

The patient was posted for laparotomy for unstable vitals and unremitting pain in the abdomen. Intraoperatively, 600 ml of blood-stained serous fluid was drained from the general peritoneal cavity, bilateral ovaries were replaced completely with cysts with fragile walls, and uterine size corresponded to the patient’s gestational age (Figure 1).

**Figure 2:**
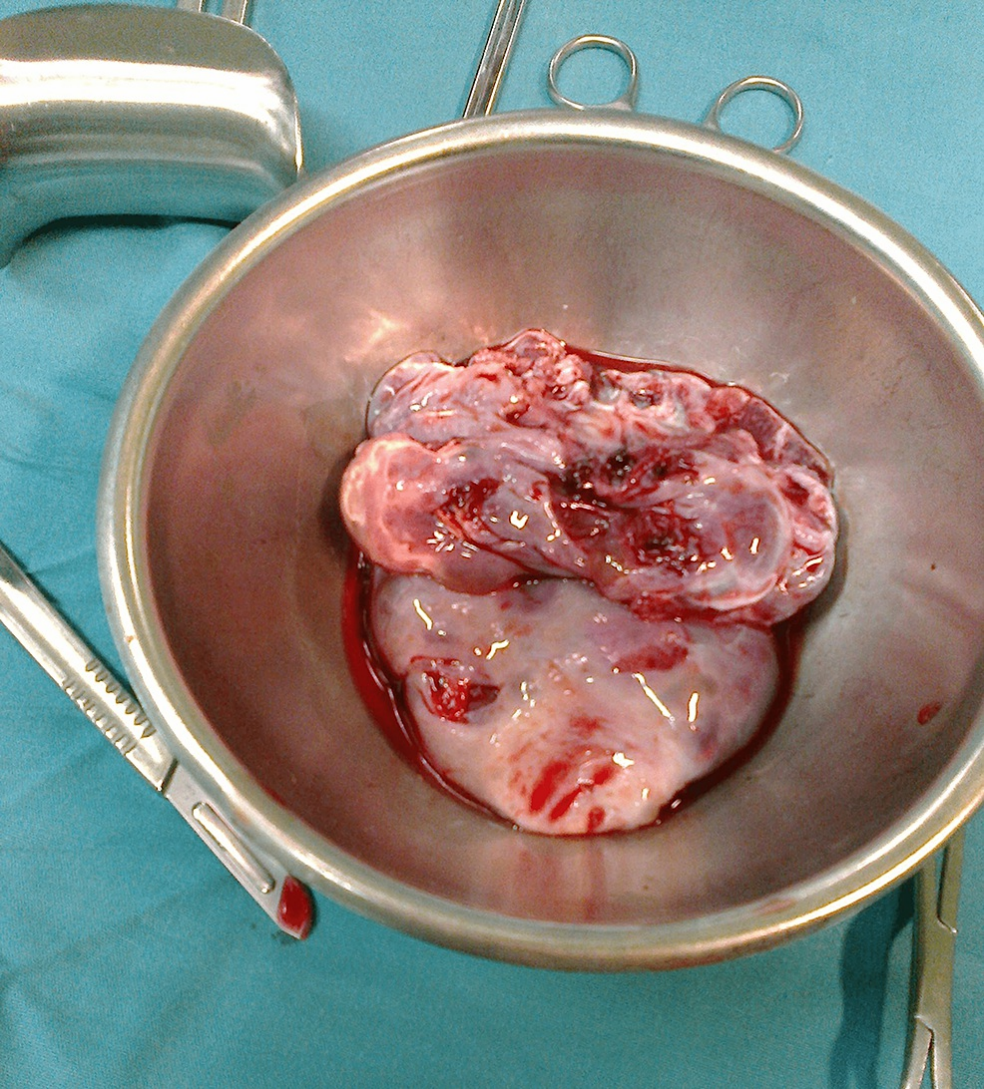
The specimen of both ovaries after partial oophorectomy was done.

Bilateral partial oophorectomy was done by conserving the residual ovaries to prevent future reproductive compromise and to protect the ongoing pregnancy (Figure 2). The histopathology report confirmed it to be bilateral theca lutein cysts.

After the surgery, the patient had regular antenatal checkups with the midwife. She had a spontaneous vaginal delivery at term gestation with an uneventful intra-and postpartum course. She delivered a female infant at a birth weight of 2850 grams with Apgar (Appearance, Pulse, Grimace, Activity, and Respiration) scores of seven and nine at one and five minutes, respectively. The patient thanked the doctors and the staff for the timely intervention and the utmost care she received.

## Discussion

Around 16% of hyperreactio luteinalis cases are diagnosed during the first trimester, and 54% are diagnosed during the third trimester or puerperium. Around 37% of patients are diagnosed during cesarean delivery [4]. A study reported an association of hyperreactio luteinalis with twin-twin transfusion syndrome and Beckwith-Wiedemann syndrome, along with multiple gestations and gestational trophoblastic diseases [3].

A case report describes a case of bilateral theca lutein cysts presenting as acute abdominal pain (torsion of one ovary) during the first 14 weeks of a singleton pregnancy. In this case, left salpingo ovariotomy and right ovarian cystectomy were performed [2]. A 30-year-old primigravida presented with hyperreactio luteinalis at 10 weeks gestation with an acute abdomen. She was managed conservatively using albumin infusion and low molecular heparin [5]. Our case is an acute abdomen presentation with rupture of bilateral theca lutein cysts in a low-resource setting that failed to respond to the conservative approach.

The maternal virilization rate has been reported to be around 30.5%. The risk of masculinization of the female fetus in hyperreactio luteinalis is decreased by placental aromatase and progesterone production in pregnancy, which have anti-androgenic properties [6]. This condition develops in response to increasing beta-human chorionic gonadotropin levels with possible increased sensitivity to circulating beta-human chorionic gonadotropin [7]. Abnormally elevated human chorionic gonadotropin levels have been linked with adverse pregnancy outcomes like abnormal fetal morphology, preeclampsia, and fetal growth restriction [6]. Our case did not have any signs and symptoms suggestive of maternal virilization, therefore we did not advise hormonal investigations.

A case report of hyperreactio luteinalis encountered during cesarean delivery described associated elevated levels of testosterone, estradiol, and beta-human chorionic gonadotropin with spontaneous regularisation to normal levels in a few months postpartum [4]. Imaging findings in hyperreactio luteinalis mimic ovarian hyperstimulation syndrome, but the latter presents with more severe symptoms, including ascites, pleural effusion, and hemoconcentration [4].

Ultrasonography is reliable in detecting the origin of the mass and in characterizing its morphology [1]. Magnetic resonance imaging (MRI) helps to differentiate hyperreactio luteinalis from ovarian malignancy, which reveals a classic 'spoke wheel’ appearance, a characteristic finding of theca lutein cysts without solid components [4]. The commonest tumor marker used for epithelial ovarian cancer is cancer antigen 125 (CA-125). It is nonspecific and can be elevated in both first-trimester pregnancy and the immediate postpartum period [1].

A multidisciplinary team of maternal-fetal medicine specialists, gynecologic oncologists, and neonatologists should ideally be consulted for adnexal masses in pregnancy, timing, and surgical treatment decisions [1]. Asymptomatic cases should be managed conservatively to avoid surgical and reproductive morbidity [6]. Ovaries in this condition return to normal size during the first few months postpartum. Recurrence of this condition has been noted in subsequent pregnancies [3]. Depending on the resources and availability of Intensive Care Unit management, we support conservative management in all non-emergent circumstances. As part of in-patient management, basic electrolytes should be measured, a complete blood count should be taken, along with multiple organ system tests, and the patient should be monitored both clinically and through laboratory testing.

## Conclusions

It is a rare finding to notice bilateral large theca ovarian cysts in the absence of a molar pregnancy and ovulation induction. Once the inciting agent is removed, conservative management shows spontaneous resolution. Surgical management is done in only selective cases if it’s associated with torsion, rupture, or hemorrhage with pregnancy. Multidisciplinary involvement is vital.
